# Variations in Soil Microbiome Assembly Determine Hatching Induction of the Carrot Cyst Nematode *Heterodera carotae*

**DOI:** 10.1111/1462-2920.70457

**Published:** 2026-10-18

**Authors:** Marine Biget, Nathan Vannier, Kévin Gazengel, Bruno Marquer, Juliette Linglin, Christophe Langrume, Catherine Porte, Sylvain Chéreau, Didier Fouville, Sylvain Fournet, Lionel Lebreton, Josselin Montarry

**Affiliations:** ^1^ IGEPP, INRAE, Institut Agro Univ Rennes Le Rheu France

**Keywords:** diacetoxyscirpenol (DAS), *Fusarium equiseti*, hatching induction, *Heterodera carotae*, soil microbiota

## Abstract

Plants shape their microbiota through root exudation to resist environmental constraints. These plant's exuded metabolites also serve as hatching signals for cyst nematodes, a major crop pest, to trigger their parasitic cycle. The “suicide hatching” strategy takes advantage of these exudates as a decoy, inducing hatching of juveniles in the absence of the host plant. We questioned here the effect of the soil microbiota on the suicide hatching induction. We used the carrot cyst nematode (*Heterodera carotae,* Jones) and evaluated the impact of three rotation crops (leek, turnip, and barley) and their associated fungal and bacterial communities on the hatching efficacy of carrot exudates in two types of soil. Our results indicated that hatching induction is lower when leek was previously cultivated. By analysing the covariation between hatching and microbiota composition, we identified fungal ASVs, notably *Fusarium equiseti,* whose abundances correlate significantly with nematode hatching. We validated that diacetoxyscirpenol, a compound produced by this species, significantly reduces nematode hatching induction. Our results demonstrated that rotation crops modulate the composition of the soil microbiome and can promote the abundance of fungi modulating the suicide hatching success. These results provide insights into plant‐microbiota‐nematode interactions for optimizing pest biocontrol strategies.

## Graphical abstract

We evaluated the impact of three soil microbiota, selected by leek, turnip, and barley, on the hatching induction of the cyst nematode *Heterodera carotae* after carrot root exudate supply. Our results demonstrated that rotation crops can promote the abundance of promising candidate fungal species modulating the hatching induction success.

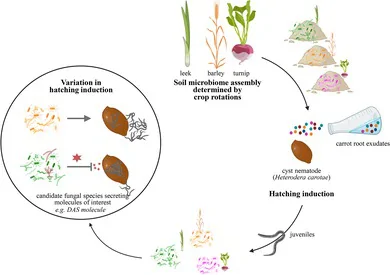

## 1 Introduction

Plant health is a major concern in agriculture, with crop yield losses due to pests and pathogens estimated at 20%–30% annually (Savary et al. 2019). Plant health is modulated by the presence and activity of various (micro‐) organisms inhabiting the surrounding soil, including viruses, bacteria, fungi and other small animals (i.e., the mesofauna) (Hacquard et al. 2015; Vandenkoornhuyse et al. 2015; Vannier et al. 2019; Topalović and Geisen 2023; Spooren et al. 2024). As sessile organisms, plants rely on soil microorganisms to cope with biotic and abiotic constraints (Müller et al. 2016; Compant et al. 2019; Ortiz and Sansinenea 2022). During their growth, plants release root exudates that modulate the composition of microbiome within the rhizosphere and, via biological selection processes, they recruit plant‐beneficial microorganisms from bulk soil, which is defined as the primary microbial reservoir (Berendsen et al. 2012; Zarraonaindia et al. 2015; Vandenkoornhuyse et al. 2015; Reinhold‐Hurek et al. 2015; Sasse et al. 2018; Luo et al. 2025). Root exudates comprise a diversity of low‐molecular‐weight organic compounds, including sugars, amino acids, organic acids, and specialized secondary metabolites like phenolics, benzoxazinoids, coumarins, glucosinolates or flavonoids (Bais et al. 2006; Badri and Vivanco 2009; Faure et al. 2009; Van Dam and Bouwmeester 2016). These compounds serve as chemical cues and nutrient sources for microorganisms in the rhizosphere (e.g., Bais et al. 2006; Badri and Vivanco 2009). This plant‐mediated microbiome assembly, together with microbes' host preference (Wippel et al. 2021) leads to a “soil conditioning effect” that can last after plant harvest (Werner and Kiers 2015; Bakker et al. 2018; Berendsen et al. 2018; Vannier et al. 2020). The soil legacy concept is directly associated with the idea of a soil conditioning effect which encompasses the effects of environmental changes (natural or human‐induced changes) on the microbial communities at a local scale, mainly driven by the root exudates (Bakker et al. 2018; Nannipieri et al. 2023). Plant root exudates therefore play a central role in structuring the soil microbiome while also acting as signalling molecules for telluric pests and pathogens to recognize their host plant (e.g., Devine and Jones 2000; Shang et al. 2000; Jones and Devine 2001; Rashid et al. 2013; Balendres et al. 2016; Zwanenburg et al. 2016).

Among the wide range of plant pests, plant‐parasitic nematodes represent an economical concern worldwide by causing important crop yield losses (Nicol et al. 2011). Among the plant‐parasitic nematodes, cyst nematodes (e.g., *Globodera* and *Heterodera* spp.) are obligatory root endoparasites (Jones et al. 2013). The carrot cyst nematode (*Heterodera carotae*, Jones) is a major threat for carrot production worldwide (see Montarry et al. 2024 for a review). The life cycle of *H. carotae* includes a survival dormant stage, the cyst, which is present in the soil and contains hundreds of eggs. *Heterodera carotae* has a very narrow host range and only infects the genus Daucus (Mugniéry and Bossis 1988). This high specialization reflects a coevolutionary process between the host and this nematode and allows the nematode to hatch only in the presence of carrot plants (Ngala et al. 2021). When perceiving carrot root exudates, second‐stage juveniles (J2) hatch from eggs and mobile J2 are released in the soil. The J2 then migrate toward the host plant and enter the roots to feed and grow (Perry et al. 2018). *Heterodera carotae* is able to complete two to four generations when a carrot is cultivated and when the climatic conditions are suitable (Brandonisio and Greco 1986; Subbotin et al. 2010; Shubane et al. 2021). Since the withdrawal of the last efficient nematicide (1,3‐dichloropropene) in Europe and with no commercial resistant cultivar, the carrot sector has faced a dramatic loss in production (Montarry et al. 2024).

Various strategies for managing cyst nematodes have been proposed, including crop rotation and intercropping, organic soil amendments, solarization, resistant plant varieties, and biological control agents, though their effectiveness varies (Ochola et al. 2021; Pires et al. 2022; Ayaz et al. 2024). One promising biocontrol strategy of cyst nematodes is the “suicide hatching”, based on the root exudates emitted by the host plant (Devine and Jones 2000). The suicide hatching strategy relies on exogenous application of host root exudate in the absence of host, which induces the hatching of J2 that cannot survive without the host (Devine and Jones 2000; Jones and Devine 2001). The effectiveness of this method has been successfully demonstrated on *Globodera rostochiensis*, *G. pallida* and *H. carotae* (Devine and Jones 2000; Jones and Devine 2001; Ngala et al. 2021; Gautier et al. 2021). Recently, a study also reported a decrease in nematode density in agricultural field trials for *G. pallida* (83% decrease) and *H. carotae* (54% decrease) using the suicide hatching strategy (Ngala et al. 2024). Therefore, this biocontrol method needs to be optimized, as previous studies have only been conducted on bare soils (e.g., Ngala et al. 2024) and have not considered the potential effect of the soil microbiota, with the exception of Gautier and colleagues (Gautier et al. 2021), that demonstrated an effect of the microbiota at a low dose of root exudates. Considering that soil microorganisms also interact with the root exudates; we propose that they may play an important role in suicide hatching of cyst nematodes in a context of plant‐microbe‐nematode interactions.

The aim of this study was to explore the impact that soil microbiota may have on hatching induction of *H. carotae*. We hypothesized that (i) the efficacy of the suicide hatching strategy is influenced by the agricultural practices (including rotation crop types, doses of the carrot root exudates, and conditions of applications) and that (ii) variations in hatching induction are influenced by specific microbial communities recruited by the rotational crops. To test these hypotheses, we first conducted a controlled pot experiment using leek, turnip, and barley, three rotation crops commonly grown between two carrot productions, to first condition the soil. We then evaluated the success of suicide hatching in two soil types, with two doses of root exudates, and at two application conditions to assess factors affecting efficiency. We analysed *H. carotae* hatching variations across these conditions and investigated the role of soil bacterial (rpoB gene) and fungal (ITS regions) communities in determining the hatching rate. Our results indicated a higher hatching induction when barley was previously cultivated, whereas lower hatching induction was observed after leek cultivation. Moreover, the hatching induction was higher in soil planted with the rotation crops than in bare soil. These findings highlighted significant variations in suicide hatching efficiency based on the rotation crop, which we partially elucidated through microbial composition analyses. Notably, we identified a candidate fungus, *Fusarium equiseti*, which was more abundant in the leek community and may impede the suicide hatching strategy.

## 2 Materials and Methods

### 2.1 Biological Materials

To test the effect of the microbiota on the hatching induction success of root exudates and to identify the best positioning of the biocontrol solution, we set up a pot experiment under controlled conditions (see the following paragraph and Figure 1) using the following biological materials:

**FIGURE 1:**
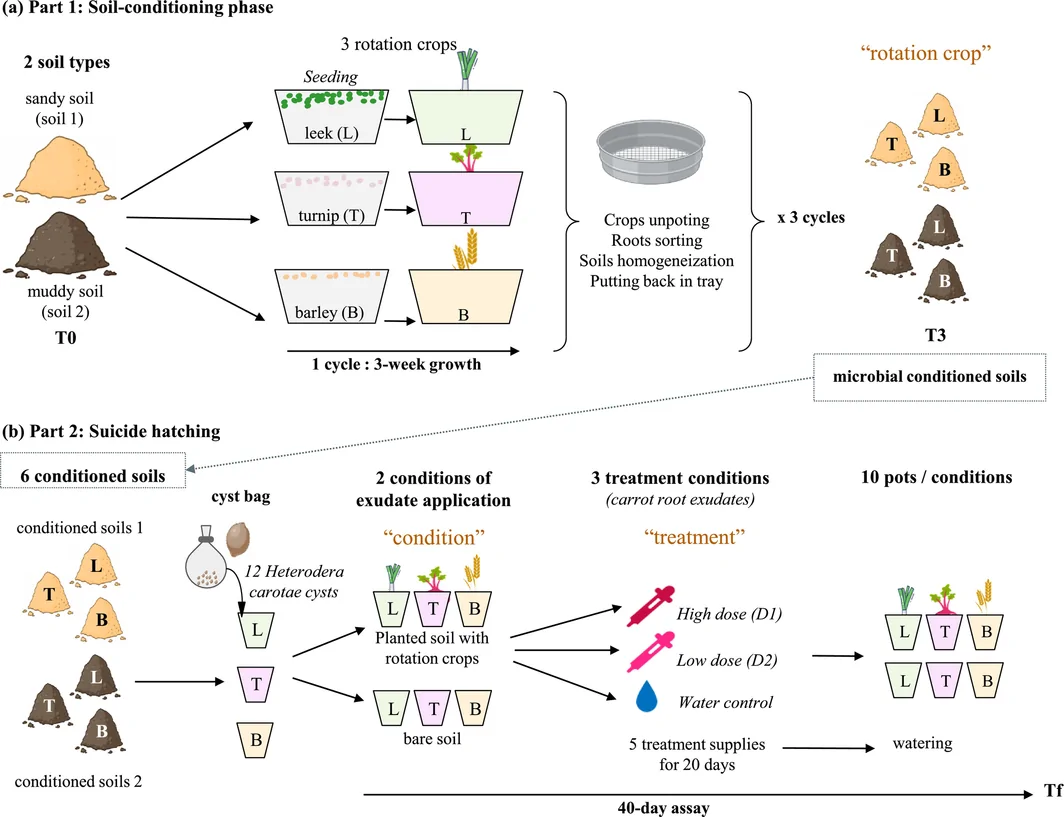
Experimental design of soil legacy and hatching suicide assays. This study comprised two main parts: Soil‐conditioning phase driven by crop root exudates (a) and the suicide hatching (response phase with the response measured on the cysts) in which the carrot root exudates are supplied (b). (a) Three crops that are commonly in rotation with carrots: the leek (L), barley (B), and turnip (T) were grown in two types of soil, the sandy (soil 1) and the muddy (soil 2) soils. The rotation crop growth lasted 3 times of 3 weeks, in three replicate trays, to ensure a shift in soil microbial communities. At the beginning (T0) and at the end (T3) of crop rotations, soils were sampled for microbial analysis. At T3, soils were collected from the trays, sieved, homogenized, and distributed into smaller pots for the second part of this study. (b) Each pot, filled with one of the six conditioned soils, contained a 12‐cyst bag to test the hatching of *Heterodera carotae* second‐stage juveniles (J2) according to the conditions. Indeed, in the pots, either the same crop (B, L or T—planted soil with rotation crops) as in the first part of the experiment was grown for 40 days, or the soil was left bare for the same period. During 20 days, carrot exudates or water (control) were applied every 4 days at two doses, a high dose (D1) or a low dose (D2), followed by a final 20‐day period of watering. Six soils out of the 10 pots were sampled at Tf for each condition for microbial analyses. The cyst bags were removed from the 10 replicates at Tf, and the J2 juveniles remaining in the cysts were counted in the laboratory as a hatching proxy (for the suicide hatching induction analyses).

#### 2.1.1 Soils Sampling and Physicochemical Analysis

Two typical soils of the carrot growing area of western France (Normandie) were used in the experiment to cover most soil types commonly found in the region of interest (coordinates of the two fields: 49°11′41.7″N 1°35′13.0″W (soil 1) and 49°14′29.1″N 1°34′39.4″W (soil 2)). This region is one of the main carrot producers in France and is facing severe infections by the carrot cyst nematode. Soil 1 is a sandy soil while soil 2 is a muddy soil and both are largely composed of sandy particles (Table S1a).

The physicochemical parameters of the soils were assessed, including pH, nitrogen N, organic carbon C, total limestone CaCO_3_, phosphorus P, particle size and conductivity. These characterizations were conducted at the beginning of the experiment (T0), after the soil conditioning phase driven by crop root exudates (T3) and after the suicide hatching assay (Tf) (Table S1b). All the measurements were performed by the Soil Analysis Laboratory LAS (INRAE, Saint‐Laurent‐Blangy, France).

#### 2.1.2 Rotation Crops

Three crop species (subsequently named “rotation crop”), commonly cultivated in rotation with the carrot ( *Daucus carota* subsp. *sativus*), were selected based on socio‐economic criteria (e.g., crop rotation, economic interest): the leek ( *Allium ampeloprasum* ; Laston F1 hybrid variety; Nunhems), the turnip ( *Brassica rapa* subsp. *rapa*; cv. Aramis; HMclause), and the barley ( *Hordeum vulgare* ; RGT planet variety; RAGT).

#### 2.1.3 Collection of Cyst Nematode and Carrot Root Exudates

Cysts of *Heterodera carotae* were extracted from soil sampled near the location where the two soils were collected (Normandie, France), using a Kort elutriator followed by a two‐step centrifugation procedure. The centrifugations allowed recovery of cysts in the supernatant. Cysts were sieved between 250 and 350 μm diameters. Twelve size‐selected cysts were then grouped in a tulle bag (150 μm stitch) to produce cyst bags for the hatching assay.

Carrots (cv. Touchon) were grown for 7 weeks in a soil‐sand mixture (1:3 sand: soil ratio), following Greco et al. (1982). The soil was then leached with deionized water to recover root exudates. The carrot root exudates were carbon dosed using a vario MICRO cube elementar analyser (Elementar GmbH, Hanau, Germany): the total carbon content was 46.7 mg of C per g of dry matter. The hatching induction of these exudates (root treatment used in Part 2) was validated by an in vitro hatching assay on *H. carotae* (average hatching rate = 76.61% ± standard error of the mean (SEM) = 15.86; data not shown).

### 2.2 Experimental Design of Soil Legacy and Hatching Suicide Assays

#### 2.2.1 Part 1—Soil Conditioning Phase: Rotation Crops Grown in Pots (Figure 1a)

Both soils were individually homogenized and split into three equal batches (for growing the leek, barley and turnip rotation crops). Each batch was sampled for microbiota analyses (T0). Three trays (27 × 32 × 17 cm) per soil and per rotation crop were then filled with around 20 kg of soil and incubated in a climate chamber where the temperature and humidity were monitored (wireless sensors LoRaWan RHF1S001—Rising HF). The soils were moistened with a nutritive solution overnight (Hoagland 1×) and the three different rotation crops were seeded at a specific density according to previous tests: 0.70 g/cm^2^ for leek, 0.52 g/cm^2^ for turnip and 0.17 g/cm^2^ for barley. Thirty‐six trays were incubated in total (2 soils × 3 crops × 6 trays). The climate chamber was calibrated at 22/16°C day/night with 16 h photoperiod for 5 days (seedling emergence) and decreased at 19/16°C day/night with 16 h photoperiod for the rest of the crop growth cycle (photoperiod sensors MCF‐LW12VOC—Engiko). The crops were regularly watered from above with a sprayer during the experiment. Soil temperature and humidity were monitored (WatchDog 1400 Micro Sensor Station, WaterScout SM100 Soil Moisture Sensor, External Soil Temperature Sensor—Spectrum technologies). After the first growth cycle (3 weeks), all crops were uprooted, and soils were subsequently sieved to remove all remaining root particles and for homogenization. Soils were then replaced in the trays before a new cycle of 3 weeks. This process was repeated two more times, for a total of three consecutive growth cycles (time‐points T3), to allow the establishment of representative microbial communities.

#### 2.2.2 Part 2—Response Phase: Suicide‐Hatching Assays (Figure 1b)

After 3 cycles of crop growth (T3), each soil was kept as a specific soil‐microbiota combination (e.g., soil 1‐Leek: sandy soil in which leeks had grown). Individual pots (7 × 7 × 13 cm) were filled with soils of the 6 soil‐microbiota combinations. The bags with 12 cysts of *H. carotae* were placed at one‐third depth of the pot and pots were placed in climate chamber. The carrot root exudates were applied on the soil surface of the pots of the two application conditions: pots planted with the three previous rotation crops (12, 9 and 3 seeds per pot for the leek, the turnip and the barley respectively) or left with bare soil. For each of these conditions, three treatments were tested: carrot exudate supplied at a high (23.3 mgC/g of dry matter) (D1) and a low dose (10‐fold diluted) (D2) and a water control. During the 40 days of suicide hatching experiment, 15 mL of root exudates or water control were supplied five times every 4 days followed by five applications of water (10 mL) until the end of the experiment to prevent plant and soil from drying out. In total, the experimental design consisted of 360 pots (6 soil‐microbiota × 2 conditions of application × 3 treatments × 10 pots). At the end of the suicide hatching assay (Tf), bags containing the 12 *H. carotae* cysts were retrieved from pots, washed in water and individually stored at 4°C in 200 μL of water to maintain ambient humidity until final population (Pf) counting (*Pf: final population—Final number of J2 remaining in the cysts*). The soil of each pot was cleaned from root debris and homogenized to sample soil for microbiota and physicochemical analyses (Tf).

### 2.3 Hatching Assays on Carrot Cysts and Calculation of Hatching Rates

#### 2.3.1 Initial J2 Content Per Cyst

Initial viable J2 content per cyst (*Pi: initial population*) was estimated at 80 viable J2 per cyst (mean = 80.6 ± standard error of the mean (SEM) = 34.3) using sized *H. carotae* cysts (250–350 μm). Briefly, 50 sized cysts were randomly selected among a pool and individually crushed in water. J2 were manually counted under binocular microscope to estimate the J2 content per cyst.

#### 2.3.2 Final J2 Content Per Cyst

Each individual cyst bag was opened at the end of the experiment, and the 12 *H. carotae* cysts were crushed to count the total number of remaining (unhatched) viable J2 (Pf). J2 remaining in cysts or hatching rate ((Pi‐Pf)/Pi) were used as proxies for hatching induction. The fewer J2 remain in the cysts, the higher the hatching rate is.

### 2.4 Characterization of Microbial Communities

#### 2.4.1 Soil Sampling and DNA Extraction

Microbial analyses were conducted on soils collected in six replicates from T0 (36 samples), T3 (36 samples), and Tf (216 samples). The 288 samples were stored at −20°C before freeze‐drying for 91 h. DNA extractions were performed from around 250 mg of dried and ground soil using the DNeasy PowerSoil Pro Kit (Qiagen) according to the manufacturer's instructions. The quality and quantity of the DNA samples were estimated using a Nanodrop, and samples were stored at −20°C until further processing.

#### 2.4.2 PCR Amplification and Amplicon Sequencing

All 288 extracted DNA samples were diluted to around 10 ng/μL to ensure similar PCR amplifications. Fungi were amplified using the *ITS* gene fragments with fungal primers ITS1‐F (5′‐CTTTCCCTACACGACGCTCTTCCGATCTGGTCATTTAGAGGAAGTAA‐3′) and ITS2‐R (5′‐GGAGTTCAGACGTGTGCTCTTCCGATCTTTYRCKRCGTTCTTCATCG‐3′). Bacteria were analysed by amplifying the *rpoB* gene fragment using the primers rpob‐F (5′‐CTTTCCCTACACGACGCTCTTCCGATCTGGYTWYGAAGTNCGHGACGTDCA‐3′) and rpob‐R (5′‐GGAGTTCAGACGTGTGCTCTTCCGATCTTGACGYTGCATGTTBGMRCCCATMA‐3′) (Ogier et al. 2019). The two sets of primers were customized to include a bridge allowing insertion of Illumina adapters and sample tags in a second PCR. Between the two PCR steps, the quality and quantity of the amplicons were estimated using the QIAxcel instrument (Qiagen) and the NanoPhotometerN120 (Implen). PCR1 and PCR2 amplicons were purified using a 0.7× ratio of magnetic beads (CleanNGS beads; CleanNA). Parameters of the PCR amplifications are detailed in Table S2a,b. The equimolar bacterial and fungal amplicon pools (50 nM) were then transferred to the EcogenO platform (Rennes, France) for quality control (FragmentAnalyzer), quantification (Kapa Library Quantification Kit‐Illumina), and sequencing (Illumina MiSeq PE‐2x250 and PE‐2x300 cycles for the fungi and the bacteria, respectively). Negative controls were included at each critical step (DNA extraction, PCR1—target amplification and PCR2—library production) and sequenced.

#### 2.4.3 Raw Data Processing and Taxonomic Affiliation

After sequencing, approximately 8600 to 57,500 reads and 2600 to 100,000 reads per sample were obtained for the ITS and the rpoB targets, respectively. A custom pipeline, including dada2 v1.14.0 R package (Callahan et al. 2016) for identification of amplicon sequence variants (ASVs) and FROGS v4.0.1 pipeline (Escudié et al. 2018; Bernard et al. 2021) for amplicon processing was used. An average of 21,631 and 47,844 reads per sample were thus retained after filtering (with no ambiguous base and a minimum read length of 100 bp) for the ITS and the rpoB target using dada2 package, respectively. After a merging step with dada2, an average of 20,074 ITS and 15,613 rpoB reads were obtained. Chimera and low‐abundance read (with an abundance < 0.005%) were then removed using FROGS. After taxonomic assignment regardless of similarity using BLASTn and NCBI nt database (Camacho et al. 2009), the final matrices contained 489 ASVs for ITS and 2552 ASVs for rpoB.

### 2.5 In Vitro Characterization of the Effect of Root Exudates From Rotation Crops and of the DAS Molecule on *Heterodera carotae* Cysts

The effect of leek, turnip, and barley exudates as well as the effect of a compound (diacetoxyscirpenol—DAS, a molecule produced by *F. equiseti* and responsible for alteration in nematode motility (Nitao et al. 1999, 2001)) on *H. carotae* hatching induction were tested using in vitro assays. Rotation crop exudates were obtained as described above for the carrot root exudates. A commercial solution of DAS (standard solution from Libios, Lyon, France) was diluted to 25 μg/mL in 0.5% DMSO according to Nitao and colleagues (Nitao et al. 2001).

Each treatment was replicated 12 times (12 cysts), with one cyst on an adapted 150 mm sieve in each well of a 12‐well plastic plate. Plates were incubated in the dark at 20°C, and the cysts were transferred to new plates at all freshly supplied solutions/exudates. At the same time, the number of hatched J2 was counted, and cumulated hatched juveniles were computed for each condition (mean of the 12 cysts). At the end of the assay, each cyst was opened to count the number of viable but unhatched J2 remaining in the cyst and to calculate the hatching rate (percentage of hatching of the viable eggs).

The different combinations of rotation crop exudates were supplied either sequentially or at different ratios. Wells were filled with 1.6 mL of the different combinations of rotation crop exudates (sequential or mixed supplies of exudates), water (negative control), and carrot exudates (positive control). For the sequential supply assay, leek, carrot, or water exudates were supplied for the first 4 days and freshly replaced with leek, carrot, or water every 4 days for 28 days, depending on the combination. The different sequential combinations of exudates tested were: leek‐carrot; carrot‐leek; leek‐leek; water‐carrot; carrot‐water. Regarding the mixed supply assay, mixes of leek, carrot, or water were supplied in different proportions every 4 days for 28 days: leek 10%‐carrot 90%; leek 90%‐carrot 10%; leek 50%‐carrot 50%; leek 100%.

For the DAS assay, wells were filled with 1.6 mL of DAS (+ carrot exudate), water (negative control), carrot exudates (positive control), and another specific control (carrot exudate + DMSO) to exclude the potential interference between DMSO and carrot exudates. Solutions were freshly supplied every 7 days for 28 days for the DAS assay.

### 2.6 Statistical Analyses

#### 2.6.1 Hatching Analyses

Statistical analyses were performed for each soil separately using the R software version 4.3.1 and the RStudio software version 2025.9.2.418. In both soils, the effects on hatching induction of the factors “treatment” (high dose, low dose or water as control), “rotation crop” (leek, turnip or barley) and “condition of application” (bare soil or soil planted with the rotation crop) nested under “rotation crop”, but also interactions among two factors, were tested using a Linear Model (function lm, “stats” package), with parametric analysis of variance (ANOVA) provided by the R package “car” (Fox and Weisberg 2018). The significance of each term in the model was determined by a Wald test and pairwise comparisons of estimated marginal means (EMMeans) were performed (function “emmeans” from the “emmeans” package) (Lenth 2019), applying the false discovery rate (FDR) correction for *p*‐values (Benjamini and Hochberg 1995).

Data of in vitro hatching assays were analysed using a Kruskal‐Wallis non‐parametric test followed by a pairwise comparison (Dunn test with FDR corrections for *p*‐values).

#### 2.6.2 Microbiome Analyses

##### 2.6.2.1 Microbial Composition

For subsequent analyses, the sequence data were first filtered. Only the samples with at least 2000 reads and the taxa present in at least 10 samples with more than 15 reads were kept. For each sample, taxa relative abundances were calculated and the composition of bacterial and fungal phyla were represented using barplots (“ggplot2” package) (Wickham 2016).

##### 2.6.2.2 Beta Diversity

Partial principal coordinate analysis (PCoAs) was performed on filtered sequence data to compare soil microbiota composition between samples. PCoAs were based on Bray‐Curtis distances (dissimilarity matrix) computed from ASVs matrices (vegdist function, “vegan” package) (Oksanen et al. 2019). Permutation tests (adonis2 function, “vegan” package, 999 permutations) were performed on the first PCoA factorial axes to test the effect and the contribution of each factor (i.e., “time‐points” (T0, T3) and “rotation crops” (leek, turnip, barley) solely at T3 for the Part 1); “rotation crops”, “condition of application” (bare soil, soil planted with the rotation crop) and “treatment” (high dose, low dose, water) for the Part 2, at Tf. Interactions among two factors were also tested. Pairwise comparisons were performed in the same way using the pairwise permutation MANOVA (pairwise.perm.manova function, “RVAideMemoire” package) (Hervé 2020).

##### 2.6.2.3 Correlations Between the Composition of Microbial Communities and Nematode Hatching

To test whether the composition of the microbiota in the different conditions could explain the differences observed in nematode hatching induction at high dose of exudates, we tested the correlation between PCoA coordinates and hatching values. To do so, we extracted the PCoA coordinates on the two first axes. We then used regressions based on linear models and tested with an ANOVA whether the hatching induction could be explained by the coordinates on the PCoA (i.e., the composition of the microbial communities within the sample). Whenever necessary to ensure model quality (normal distribution of residuals) the variables were normalized with a log‐transformation. Analyses were done similarly for both bacterial and fungal datasets.

##### 2.6.2.4 Microbial Differential Abundance Testing and Correlation Between Fungal Taxa and Nematode Hatching

To test whether specific ASVs could contribute to the observed variations in nematode hatching induction, we compared ASV abundances (counts) in treatment with low vs. high hatching induction. To do so we compared ASV abundance at Tf in the soil 1 at high dose of exudates between leek (low hatching induction) and barley (high hatching induction) using DESeq2 (Love et al. 2014) with a wald test and a parametric fittype. *p*‐values were adjusted with FDR. The correlation between the abundance of significantly enriched ASVs and the hatching induction was then tested across all samples of the soil 1 and high dose using regressions based on linear models and an ANOVA test. Whenever necessary to ensure model quality (normal distribution of residuals) the variables were normalized with a log‐transformation. Analyses were done similarly for both bacterial and fungal datasets.

## 3 Results

### 3.1 Suicide Hatching Induction Success Is Modulated by the Rotation Crop

The suicide hatching experiment (Figure 1) was carried out in two types of soil in which three different rotation crops (barley, leek, and turnip) were grown to obtain microbial conditioned soils (Figure 1a). Following this first part, pots were filled with one of these conditioned soils and bags of cysts of *Heterodera carotae* were added to each pot. To test the condition of treatment application, half of the pots were then seeded with either the same crop as in the first part of the experiment, or the soil was left bare (Figure 1b). Carrot exudates (treatment) or water (control) were supplied at two doses (Figure 1b). To test the effect of the dose of exudates (high dose—D1, low dose—D2, and water as control), the condition of root exudate application (“bare soil” or “soil planted” with rotation crops) and the rotation crop type (barley, leek, and turnip) on *H. carotae* hatching induction in the two soils, we analysed the number of second‐stage juveniles (J2) remaining in the cysts after root exudate application.

The mean number of J2 remaining in the cysts (a proxy of hatching induction) was similar in the water controls of both soils (soil 1 (19.9% of hatching in comparison to the Pi of *H. carotae* cysts): mean = 64.6 ± standard error of the mean (SEM) = 1.4; soil 2 (19.7% of hatching): mean = 64.7 ± 1.4) independently of the rotation crop and the condition of application (Figure 2a,b; Figure S1a,b).

**FIGURE 2:**
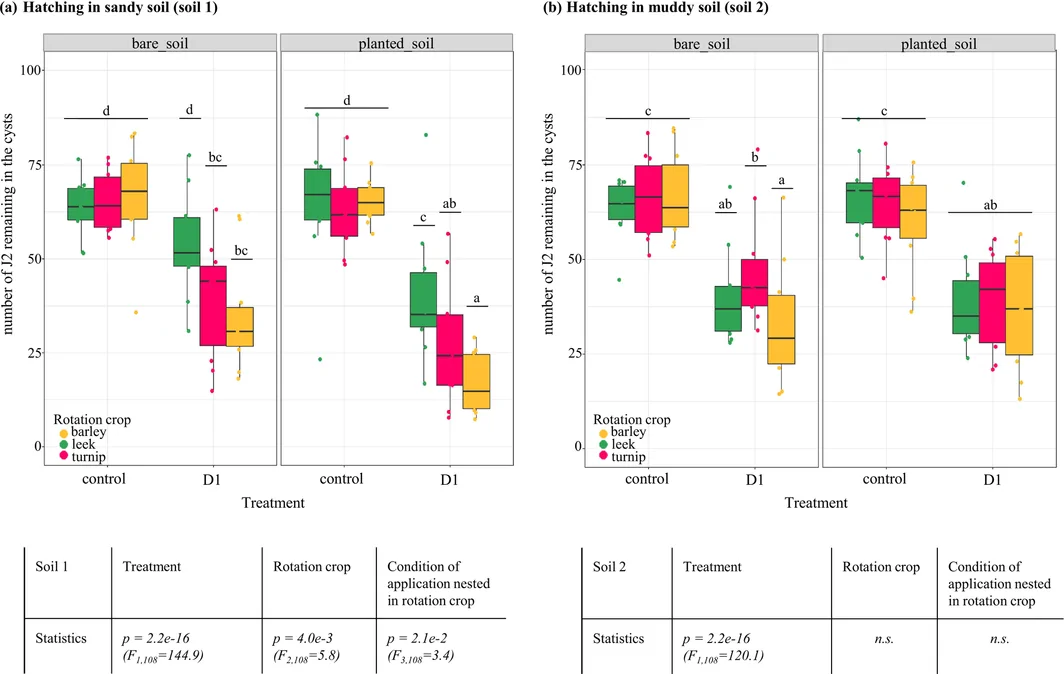
Effects of rotation crops, root exudate treatments and conditions of exudate application on hatching of *Heterodera carotae* juveniles. Boxplots representing the mean values of the J2 remaining in the cysts as a hatching proxy (mean values ± SEM) at the end of the experiment Tf (after 40 days) for the sandy soil (soil 1) (a) and the muddy soil (soil 2) (b). Two treatments are represented: Control (water) and D1 (high dose of root exudates), either for the bare soil or the soil planted with the rotation crop “planted soil” (condition of exudate application) and the three rotation crops: Leek (green), turnip (pink), barley (yellow). Letters represent significant differences among every combination for a soil (linear model with the factor “condition of application” nested in the factor “rotation crop”). Statistical values of the models are summarized below the boxplots—Treatment (soil 1: *p* = 2.2e‐16; soil 2: *p* = 2.2e‐16); Rotation crops (soil 1: *p* = 4.0e‐3; soil 2: n.s.); Condition of application (soil 1: *p* = 2.1e‐2; soil 2: n.s.).

The application of carrot root exudates significantly stimulated the hatching of *H. carotae* J2 in both soils as there was a significant decrease in the number of J2 remaining in the cysts compared to the water control (“treatment” factor: soil 1: *F*_1,108_ = 144.9, *p* = 2.2e‐16—Figure 2a; soil 2: *F*_2,108_ = 120.1, *p* = 2.2e‐16—Figure 2b). In addition, a significant dose effect of carrot exudates was observed with respectively 25.7% and 82.0% increase in hatching rate in the low and high dose treatment compared to the water control (soil 1—D2: mean = 51.4 ± 2.3; soil 1—D1: mean = 35.5 ± 2.4 (Figure S1a), while for soil 2—D2: mean = 54.7 ± 2.5; soil 2—D1: mean = 39.1 ± 1.9 (Figure S1b)). In soil 1, the rotation crops and the condition of application (soil planted with rotation crops “planted soil” or bare soil) significantly impacted the hatching induction (“rotation crop”: *F*_2,108_ = 5.8, *p* = 4.0e‐3 and “condition” factor nested in “rotation crop”: *F*_3,108_ = 3.4, *p* = 2.1e‐2, respectively—Figure 2a) with higher hatching induction in the presence of the rotation crop compared to the bare soil (soil 1—D1—bare soil (47.1% of hatching): mean = 42.6 ± 3.0; soil 1—D1—“planted soil” (64.9% of hatching): mean = 28.3 ± 3.2 (Figure 2a), and for D2, soil 1—D2—bare soil (28.5% of hatching): mean = 57.6 ± 3.3; soil 1—D2—“planted soil” (43.9% of hatching): mean = 45.2 ± 2.7 (Figure S1a)). Interestingly, the type of rotation crop also altered the hatching, with lower hatching induction when leek had been previously grown (49.2% of hatching; D1, “planted soil”: mean = 40.6 ± 5.7 (Figure 2a)). Conversely, a higher hatching was observed with the barley as rotation crop, especially in the pots planted with rotation crops at high dose of exudates (D1, “planted soil” (79% of hatching): mean = 16.9 ± 2.6 (Figure 2a)). Contrary to the results in soil 1, no significant differences were observed for the condition of application nor the rotation crop in soil 2 (Figure 2b; Figure S1b). These results demonstrated the efficacy of carrot root exudates on *H. carotae* and highlighted the existence of soil‐, rotation‐, and condition‐dependent hatching induction success.

### 3.2 Soil Physicochemical Properties or Addition of Rotation Crop Exudates Did Not Affect Hatching of *H. carotae*

To investigate the impact of two soils on *H. carotae* hatching induction, we first compared the physicochemical properties of the soil samples collected at the beginning of the experiment (T0), after the soil conditioning phase in the two soils (T3) and after root exudate application (Tf) (Table S1a,b). Regardless of the sampling time‐points, the physicochemical properties significantly differed between the two soils, although initially they already differed in texture (e.g., clay: 25 g/kg in soil 1 vs. 117 g/kg in soil 2; silt (mean of coarse and fine silt values): 10 g/kg in soil 1 vs. 178 g/kg in soil 2; sand (mean of coarse and fine sand values): 965 g/kg in soil 1 vs. 705 g/kg in soil 2; (Table S1a)), and specific parameters such as organic matter (OM: 8 g/kg in soil 1 vs. 28 g/kg in soil 2), organic carbon content (TOC: 4.6 g/kg in soil 1 vs. 16.2 g/kg in soil 2), limestone (CaCO_3_: 108 g/kg in soil 1 vs. 19 g/kg in soil 2) or nitrogen (N: 0.4 g/kg in soil 1 vs. 1.7 g/kg in soil 2) (Table S1b). However, rotation crop, condition of root exudate application (bare soil or soil planted with rotation crops) and root exudate treatment (high dose, low dose or water control) did not affect soil physicochemical properties (Figure S2). Thus, the observed variations in hatching induction cannot be ascribed to the soil physicochemical properties.

Then, we tested whether the observed effect of the three rotation crops on *H. carotae* hatching could be attributed to their exudates modulating the chemical signal of the carrot exudates. We conducted in vitro hatching assays including sequential and mixed addition of rotation crops and carrot exudates. The hatching assays revealed that none of the exudates from leek, turnip, or barley induced the hatching of *H. carotae* J2 juveniles (data not shown), and neither a sequential application of leek and carrot exudate (Figure S3ai,ii; Table S3a) nor a mixture of these exudates (Figure S3bi,ii; Table S3b) interfered with hatching. Collectively, these results confirm that the observed variations in hatching induction did not depend on soil's physicochemical properties nor a direct interaction with molecules present in the exudates of the rotation crops.

### 3.3 Rotation Crop Structures Bacterial and Fungal Communities

Because neither the rotation crop exudates nor the soil physicochemical properties can explain the observed variations in *H. carotae* hatching, we explored the role of the soil microbiome as a modulator of nematode hatching. First, we showed that the bacterial and fungal communities were significantly different between the two types of soils (Figure S4a,b). Indeed, the factor “soil” accounted for 56.6% and 61.5% of the variance in bacterial and fungal assemblages respectively (Figure S4a,b). Consequently, we tested for each soil independently the effect of the rotation crop, the time‐point of sampling, the condition of root exudates application, and the dose of exudates on the structuration of the microbial communities. To do so, we characterized soil bacterial and fungal communities at T0, T3, and Tf using amplicon sequence variants (ASVs).

#### 3.3.1 The Microbial Community Composition Is Shaped by Rotation Crop

The relative abundances of bacteria and fungi at the phylum level did not differ between T0 and T3 in the two soils (Figure S5a,e for the bacteria and fungi, respectively). Pseudomonadota (mostly represented by Alphaproteobacteria at the class level; Figure S5di–iii) and Actinomycota dominated the bacterial communities in all the samples at T0 and T3 (e.g., the mean relative abundance of Pseudomonadota in soil 1 was 0.50 and 0.57 in soil 2 (Figure S5a)). The bacterial relative abundance patterns did not differ at Tf compared to T0 and T3 (Figure S5b,c). The Ascomycota were the most abundant fungal taxon at T0, T3 and Tf, although slight differences were observed for the two soils planted with the rotation crop turnip (e.g., Tf; soil 1, soil 2: mean = 0.60) (Figure S5e–g). At the class level, the Sordariomycetes constituted the greater part of the Ascomycota; (Figure S5hi–iii).

The influence of the different factors on microbial community structure was analysed through principal coordinate analysis (PCoA—Bray Curtis distances) at Tf (Figure 3; Figure S6). The conditions of exudate application had no effect on the bacterial community (Figure S6a,b), while it slightly explained a fraction of the fungal community composition (Figure S6c,d). PCoA analyses and permutation tests revealed a significant effect of the rotation crop for bacteria (*p* = 1.0e‐3—Figure 3a–d) and fungi (*p* = 1.0e‐3—Figure 3e–h) regardless of soil type. The rotation crop accounted for 12.0%, 18.7%, 9.7% and 10.0% of the variance in bacterial assemblages for the soil 1 ‐bare soil, ‐planted soil and soil 2 ‐bare soil, ‐planted soil respectively (Figure 3a–d; Figure S6a,b). Regarding the fungal assemblages, this factor accounted for 10.4%, 23.6%, 14.0% and 49.2% of the variance for the soil 1 ‐bare soil, ‐planted soil and soil 2 ‐bare soil, ‐planted soil respectively (Figure 3e–h; Figure S6c,d). Notably, the significant rotation crop‐mediated clustering of microbial communities was also observed at earlier sampling time‐point (T3) in the two soil types (Figure S7). At T3, rotation crops explained around 20% of the variance in both microbial communities across both soil types (Figure S7a–d). Interestingly, the carrot root exudate treatment did not impact the composition of bacterial and fungal communities in both soils (Figure 3a–h; Figure S6a–d). The microbial community structure was thus mainly shaped by the rotation crops and could explain the observed modulations of *H. carotae* hatching induction.

**FIGURE 3:**
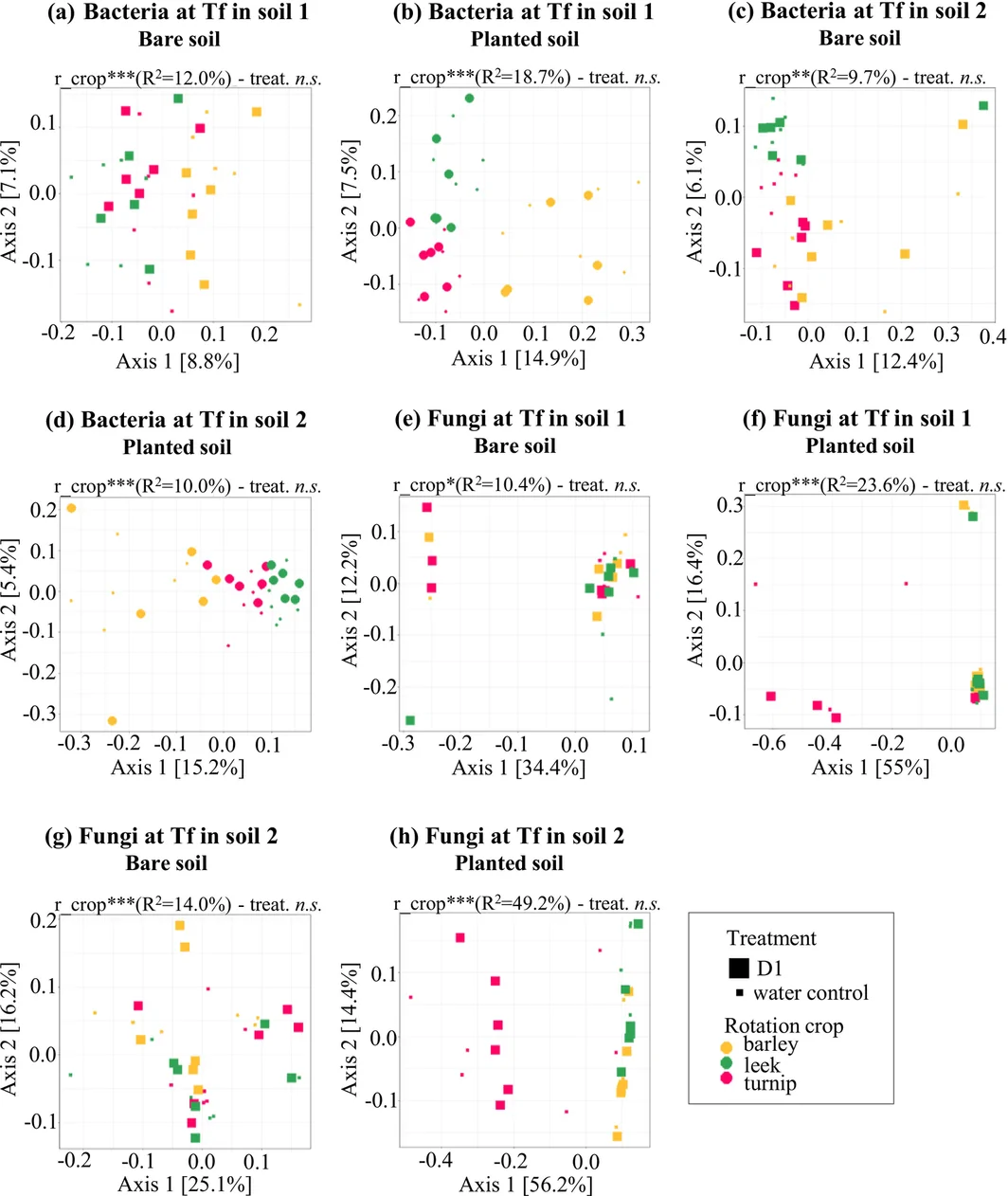
Microbial community composition is structured by the rotation crop and persists after carrot root exudate application. Principal coordinate analysis (PCoA—Bray Curtis index) plots depicted the bacterial (a–d) and fungal (e–h) communities (ASV composition) of the two soils at Tf: The sandy soil (soil 1) (a, b, e, f) and the muddy soil (soil 2) (c, d, g, h). Two treatments are represented: Control (water) and D1 (high dose of exudates), either for the bare soil (a, c, e, g) or the soil planted with the rotation crop “planted soil” (b, d, f, h) and the three rotation crops: Leek (green), turnip (pink), barley (yellow). Results from the permutation tests (permanova) testing the rotation crop (r_crop—barley, leek, turnip) and the treatment (high doses (D1), water control) effects and the *R* ^2^, percentage of variance explained, are summarized above the plots (*: *p* < 0.05; **: *p* < 0.01; ***: *p* < 0.001; ns: *p* > 0.05).

### 3.4 Fungal Taxa Were Associated With Hatching Induction Success

In order to identify whether the most contrasted variations in hatching induction (soil 1, high dose of exudates) can be explained by the changes in microbiome composition, we first tested the correlation between the overall microbiome composition and hatching induction before looking precisely at the microbial taxa level.

#### 3.4.1 Soil Microbiome Composition Partially Explains Variations in Hatching

First, we tested whether sample coordinates on the PCoA (i.e., the variation of microbiome composition between samples) could explain the observed hatching induction in soil 1 (Figure S8a,b). We found a significant correlation between number of J2 remaining in the cysts and bacterial communities (linear regression: *p* = 7.6e‐3, *R* ^2^ = 17.3%—Figure S8a) but no significant effect was detected for the fungal microbiome (Figure S8b). These results suggest that the overall microbiota composition could only explain a fraction of the variations observed in hatching induction which indicates that only specific taxa or species could be involved in the observed hatching differences in soil 1.

#### 3.4.2 Few Fungal and Bacterial Taxa Are Differentially Abundant in Contrasted Hatching Conditions and Correlate With Hatching Induction

To determine whether individual microbial taxa could be enriched or depleted between conditions in which large hatching induction contrasts were observed (i.e., soil 1, high dose of exudates D1—Barley vs. Leek—Figure 2a), we performed DeSeq2 analyses for both bacterial and fungal datasets. We thus compared the count of ASVs between leek and barley samples at high dose of exudates in the soil 1 (Figure 4). Among the 1933 bacterial ASVs tested, only a single ASV, belonging to the Pseudomonadota phylum was significantly enriched in leek compared to barley (ASV_445‐ *Georhizobium profundi*‐ *p* = 5.0e‐3; baseMean = 10.50 reads; log2FoldChange = 6.62; Figure 4a, Table S4a,b). Among the 287 fungal ASVs tested, 7 ASVs were differentially abundant in barley versus leek with 5 ASVs significantly enriched in leek (ASV_2, ASV_48, ASV_101, ASV_158, ASV_166) and 2 ASVs significantly enriched in barley (ASV_100, ASV_144) (Figure 4b, Table S5a,b). The ASV_2, more abundant in the leek condition, was the second most abundant fungal ASV in the entire dataset with an average baseMean number of reads of 1837.82 per sample (i.e., DESeq2 calculated baseMean, see Table S5a).

**FIGURE 4:**
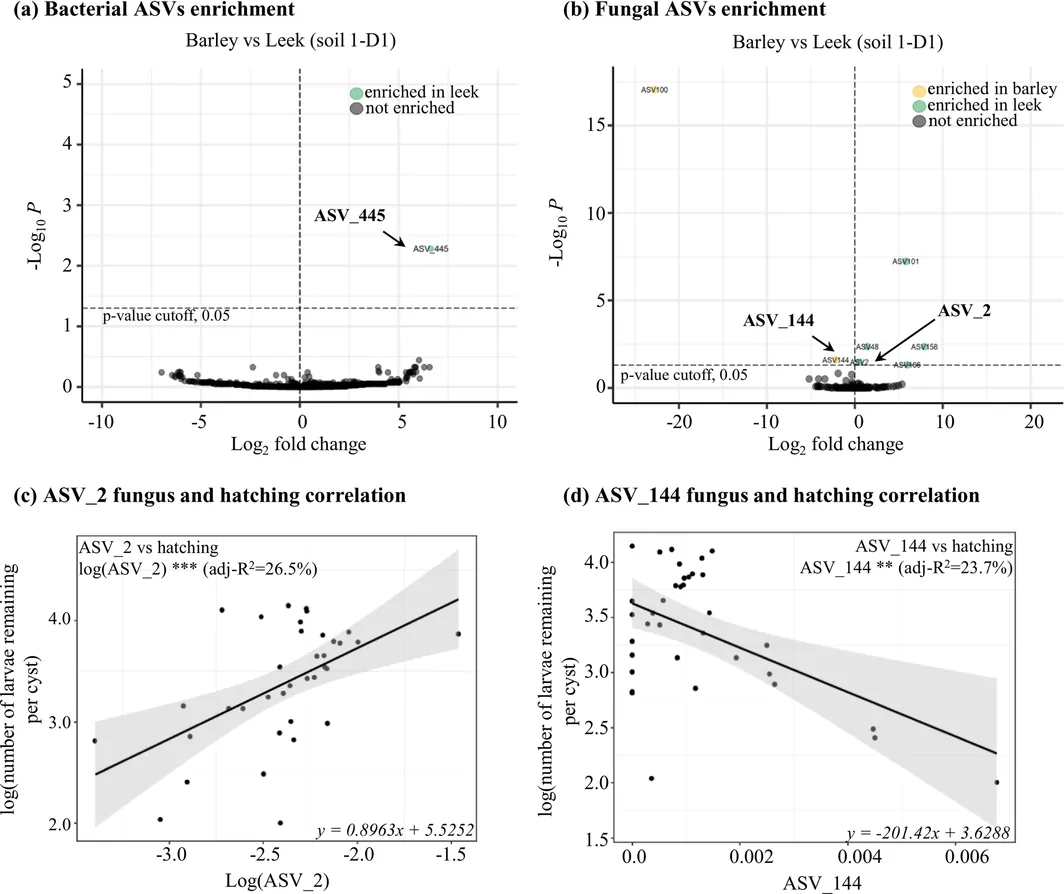
Hatching correlates with fungal ASV enrichment but not with the overall microbial composition. Volcano plots depicting differentially abundant bacterial (a) and fungal (b) ASVs in sandy soil (soil 1), at the high exudate dose (D1) between barley and leek (condition in which the largest variation in hatching induction has been observed) (DESeq2). ASVs significantly enriched in barley are highlighted in yellow and ASVs enriched in leek in green (DESeq2 Wald test with FDR adjusted *p* < 0.05). Fungal ASV_2 and ASV_144 have been pointed out as narrow due to their significant correlation between ASV abundance and hatching induction (b). Linear regression between the abundance of log(ASV_2) (c) and ASV_144 (d) and the number of larvae remaining per cyst across all high dose samples of the sandy soil (soil 1) (results were obtained from a linear model and an ANOVA; **: *p* < 0.01; ***: *p* < 0.001—equations of the two regression curves are displayed in the plots).

We further examined the correlation between relative abundances of differentially enriched ASVs and hatching induction (with the number of larvae remaining per cyst as a proxy of hatching rate) using linear regressions. The abundance of the bacterial ASV_445 did not correlate with the hatching induction (*p* = *n.s*., Table S6a). Regarding the fungal ASVs, only two ASVs (ASV_2 and ASV_144) were significantly correlated with the hatching induction (Figure 4c,d). Individually, ASV_2 and ASV_144 explained 26.5% and 23.7% of the hatching induction variance, respectively (ANOVA: ASV_2: *F*_1,34_ = 13.6; *p* = 7.9e‐4—Figure 4c, Table S6b; ASV_144: *F*_1,34_ = 11.9; *p* = 1.5e‐3—Figure 4d, Table S6c). When tested these two ASVs in a single model, their abundances explained up to 47% of the hatching induction variance (ANOVA: ASV_2 + ASV_144: *F*_2,33_ = 16.4; *p* = 1.1e‐5—adj‐*R* ^2^ = 46.9%—Table S6d). The ASV_144, that was negatively correlated with the number of larvae remaining in the cysts and therefore positively correlated with the hatching induction, could not be assigned taxonomically. Conversely, the ASV_2, that was negatively correlated with the hatching induction, was assigned to the species *Fusarium equiseti* (Tables S5a and S6b).

### 3.5 The Diacetoxyscirpenol (DAS) Produced by *F. equiseti* Inhibits *H. carotae* Hatching

We further tested the effects of DAS molecule on hatching of *H. carotae*. The DAS molecule has been previously shown to be secreted by *F. equiseti* (Nitao et al. 2001) (Figure 5a). As expected, the positive control (carrot root exudates + water) induced 98.8% of nematode hatching, while the negative control (water) induced only 2.2% of nematode hatching. In addition, DMSO used to dilute DAS did not interfere with hatching induction by the carrot root exudate (cumulative hatching of 97% after exudate addition). On the other hand, the nematode hatching was significantly inhibited by the DAS molecule (cumulative hatching of 2.9% after exudate addition (Figure 5b)). The observed reduction of 94.1% in the average hatching induction indicates that the DAS molecule is able to strongly inhibit the hatching of *H. carotae*.

**FIGURE 5:**
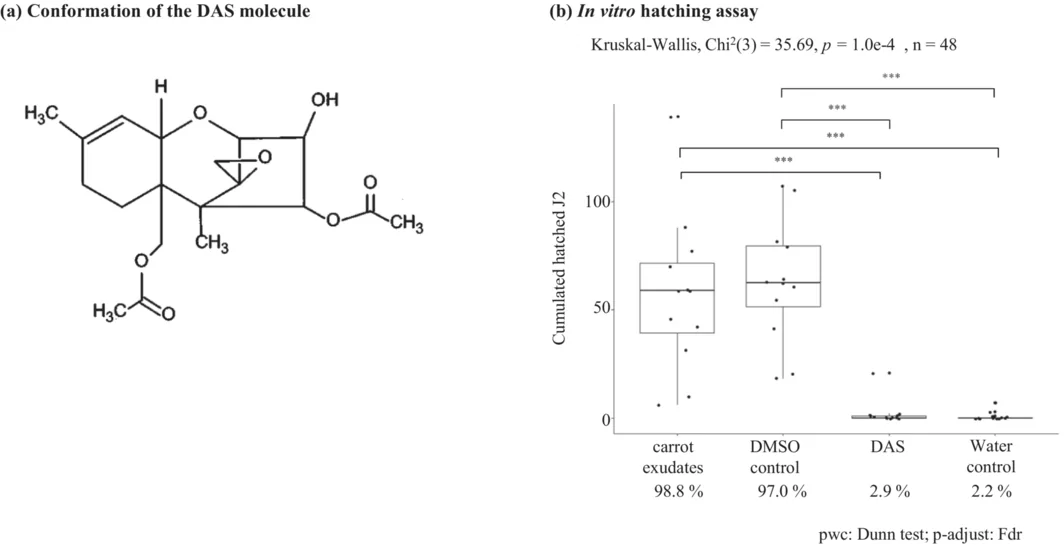
DAS inhibits the hatching induction of *Heterodera carotae*. Structure of the diacetoxyscirpenol molecule from Nitao et al. 2001 (a). Boxplots representing in vitro *H. carotae* hatching results (b) (mean of cumulated hatched J2 of 12 replicates ± SEM) depending on the tested molecule and the adequate controls. Positive control: Carrot root exudates + water; negative control: Water control; DMSO control: 0.5% DMSO used for DAS dilution; DAS molecule used at 25 μg/mL according to Nitao and colleagues (Nitao et al. 2001). Percentages of hatching are provided for each condition (number of hatched juveniles compared to total number of juveniles present in the cysts). Data were analysed using a Kruskal‐Wallis non‐parametric test (*p* = 8.7e‐8; *n* = 48) followed by a pairwise comparison (Dunn test with FDR corrections for *p*‐values). Pairwise comparisons: DAS versus DMSO_control—*p* = 6.3e‐5; DAS versus carrot root exudates (positive_control)—*p* = 2.0e‐4; water (negative_control) versus DMSO_control—*p* = 2.6e‐5; water (negative_control) versus carrot root exudates (positive_control)—*p* = 6.3e‐5.

## 4 Discussion

The suicide hatching strategy, that relies on nematode specificity to host root exudates, has emerged as an effective and sustainable management approach for controlling cyst nematodes (e.g., Ngala et al. 2021, 2024). Our work demonstrated a significant hatching induction of *Heterodera carotae* juveniles in absence of its host plant following exudates supply, with a major dose‐dependent effect. Our results highlighted that observed variations in induction success are connected to differentiation in soil microbiome composition following different crop rotations and are likely associated with the enrichment of specific microbial taxa. We further evidenced a crucial role of the soil microbiota in modulating hatching induction, with a particular emphasis on the impact of candidate fungal species and fungal‐secreted compounds associated with previously cultivated rotation crops. These findings provide valuable insights to optimize the application of biocontrol products and raise critical questions regarding the underlying mechanisms of fungal‐mediated inhibition of nematode hatching.

In the field, carrots are grown in rotation with various crops that assemble distinct rhizospheric microbiomes through exudation and mobilize essential microbial functions (Vandenkoornhuyse et al. 2015). As a result, carrots are grown in distinct environments with contrasted microbiomes depending on the preceding crop. Our results indicated a strong structuring of microbial communities depending on rotation crops: barley, turnip, or leek (Figure 3; Figure S6) that supports the existence of a soil legacy (Bakker et al. 2018; Berendsen et al. 2018). Several previous studies have already demonstrated the role of rotation crops in structuring soil microbial communities (e.g., Larkin and Honeycutt 2006; Xuan et al. 2012; Soman et al. 2017; Kracmarova et al. 2022; Yang et al. 2023). Variations in soil microbial community composition can influence plant responses to biotic stresses, such as parasites, during early development (e.g., Vannier et al. 2019; Zhou et al. 2023). Similarly to other plant species, carrot root exudates can influence the rhizosphere soil microbiome composition (e.g., Anderson et al. 2024) but nematode juveniles perceive hatching signals at an early seedling stage (Greco et al. 1982) and the microbial composition of the bulk soil is likely mainly shaped by the previous crop. The influence of crop history was previously tested experimentally and authors demonstrated that the effect of previous plant species on the soil microbiota, particularly fungal communities, outweighed the effect of the current plant (Hannula et al. 2021). This soil legacy may disrupt juvenile perception of hatching signals, potentially affecting nematode dynamics.

In our experiment, nematode larval hatching was induced by applying root exudates to lure the parasite, rather than using trap carrot crops. Despite high hatching induction in the two soils tested, the impact of rotation crops on nematode hatching induction was only observed in the sandy soil (soil 1), not in the muddy soil (soil 2). In the sandy soil, hatching was lower after leek cultivation compared to turnip and barley, hatching induction for the latter being the strongest. This effect cannot be attributed to changes in the soil's physicochemical properties, as only minor differences in the measured properties were observed at the end of the soil conditioning phase. In addition, nematode hatching was also not affected by the exudates of rotation crops, as none of the sequential nor the mixture applications of leek and carrot exudates interfered with hatching (Figure S3a,b). Instead, this effect could result from the observed shifts in bacterial and fungal communities driven by the rotation crops and even more, because the microbiota of the two soil types were also significantly different (Figure S4a,b). As previously noted by Gautier and colleagues, we could hypothesize that the microbial community selected by leek was enriched in copiotrophic species (Gautier et al. 2020). Indeed, although identified in other cyst nematodes, the hatching factors characterized to date, such as solanoeclepin A (C27H30O9), glycinoeclepin A (C25H34O7), α‐solanine (C45H73NO15), or α‐chaconine (C45H73NO14), are mostly carbon‐based molecules (see Sikder and Vestergård 2020 for a review). Therefore, the hatching factors of *H. carotae* are also most likely carbon‐based molecules, which could serve as a carbon source for soil microorganisms, especially for copiotroph microorganisms that exhibit high growth rates in response to the availability of carbon resources (Fierer et al. 2007). Thus, the carrot root exudate may have been metabolized at higher rates by the copiotrophic microorganisms selected by leek (compared to barley), blurring the nematodes' hatching signals. However, this hypothesis is not fully supported as we only found a weak correlation between the overall bacterial community composition and hatching rates and no correlation at all for the fungal community composition in the sandy soil (Figure S8a,b). To further investigate the link between overall microbial composition in different types of soils and variations in hatching induction, we also performed the same type of correlation in the muddy soil and showed significant correlations for the bacteria and fungi, while no rotation crop effect was recorded on hatching induction (Figure S8c,d, Figure 2b). Nevertheless, this does not rule out the hypothesis that leek could enrich the soil with *H. carotae*‐antagonistic microbes in the sandy soil and could explain such hatching variation depending on rotation crop.

Accordingly, we showed that taken together two fungal taxa explained up to 47% of the hatching induction variance in the sandy soil (soil 1, barley vs. leek, Figure 4). In particular, the ASV_2 belonging to *F. equiseti* was enriched during leek cultivation and its relative abundance was positively correlated with the inhibition of *H. carotae* hatching. The abundance of this candidate fungus was 4.5 times lower at Tf in muddy soil (soil 2) compared to the sandy soil (Figure S9a), which may explain the absence of a significant rotation crop effect on the hatching induction observed in the muddy soil. Similarly, the ASV_144, which was enriched during the barley cultivation and significantly correlated with the hatching induction, was also more abundant in soil 1 than in soil 2 (Figure S9b). While no taxonomic assignment was obtained with a BLAST search, we cannot exclude the hypothesis that this fungus may produce a hatching factor or compete with microbes that produce hatching inhibitors. Regarding *Fusarium equiseti*, it has been previously reported to exhibit nematicidal activity (nematostatic, nematophagous) (Haarith et al. 2020). In particular, Nitao and colleagues isolated *F. equiseti* from soybean cyst nematodes (SCN) ( *Heterodera glycines* ) and demonstrated a negative correlation between the relative abundances of this fungus and the viability of *H. glycines* and *Meloidogyne incognita* (Nitao et al. 1999, 2001). Moreover, Lopez and colleagues recently described the core fungal communities of soil samples with four levels of SCN abundance and identified *F. equiseti* as a promising candidate for SCN antagonism (López et al. 2024). Interestingly, Nitao and colleagues have purified a molecule, the diacetoxyscirpenol (DAS), from *F. equiseti* and showed that this mycotoxin inhibits hatching and motility of *M. incognita* J2 (Nitao et al. 2001). In our study, we confirmed the nematicidal potential of DAS by demonstrating its ability to inhibit the J2 hatching of *H. carotae*. Nonetheless, the precise taxonomy of the candidate ASVs identified, especially *F. equiseti*, needs to be confirmed using culture‐based approaches and genome sequencing. On the same line, further studies are required to validate its ability to produce DAS (e.g., mass spectrometry) and to inhibit *H. carotae* hatching.

Furthermore, our results suggest that applications of carrot root exudates are more efficient when supplied after crop sowing, with the exception of leek. The higher hatching observed in the presence of rotation crops could be attributed to the role of the plant root systems as facilitators for the capillary spread of exogenous exudates toward cysts (Hinsinger et al. 2009). According to our data at high dose of exudates, the nematode population could be reduced by 33% in a sandy soil with a microbiota selected by leek in the absence of the rotation crop while it could be reduced by up to 79% in a sandy soil (soil 1) with a microbiota selected by barley and in the presence of the rotation crop. In soil 2, neither the crop rotation type nor the condition of exudate application had a significant effect on hatching induction. This could explain why the range of hatching variations was smaller (from 41% to 59%; turnip—barley); nevertheless, the hatching induction remained highly effective in a muddy soil. These results, obtained in controlled conditions, need to be strengthened by performing field trials to optimize the positioning of a biocontrol product stimulating the hatching induction of *H. carotae* and to confirm hatching induction success in other soil types. Our results also suggested that a crop rotation system, including leek just before a carrot crop, could reduce the hatching of *H. carotae*. While the temporal diversification was tested in our study (i.e., crop rotations), a spatial diversification including crops that modulate cyst hatching could also potentially have a positive impact. An interesting prospect would be to alternate rows of leek and carrot in cultivated plots to determine whether it decreases *H. carotae* damages.

Finally, our study raised important questions in microbial ecology research, especially regarding the processes and dynamics of the tripartite interaction (plant‐microbiota‐pathogen) beyond the transitory dysbiotic state of the carrot holobiont (Arnault et al. 2023). Beyond direct interactions with pathogens and pests, the plant microbiota also contributes to the modulation of plant immune responses (Vannier et al. 2019; Ma et al. 2021; Ordon et al. 2025). Whether the observed variations in microbiota composition modulate the host immunity in the field remains to be tested. While *Fusarium* species are well‐known plant pathogens responsible for various plant diseases (Beccari et al. 2022), here we demonstrated their possible counter‐balancing role in preventing nematode attack. Accordingly, the protective capacity of nonpathogenic strains of *Fusarium oxysporum* was previously documented (see Alabouvette et al. 2009 for a review). Conceptually, *Fusarium* species should be considered not only as soil‐borne pathogens but also as part of the microbiota and consequently as a functional constituent of the carrot holobiont (Theis et al. 2016). Our results strengthen the emerging idea that the notion of pathogen should be scaled up at an ecosystem level, as stated in the pathobiome concept (Bass et al. 2019), by considering that any changes affecting the ecosystem functioning can balance the tripartite interaction and determine plants' health.

To conclude, our study suggests that modifying agricultural practices can improve the success of hatching induction strategies while also influencing pest populations by affecting the soil microbiota. This highlights the importance of soil microbial communities in sustainable agriculture and emphasizes the need to systematically consider microorganisms in the analysis and understanding of field diseases.

## Author Contributions

**Marine Biget:** investigation, writing – original draft, formal analysis, methodology, visualization, software. **Nathan Vannier:** software, formal analysis, writing – original draft. **Kévin Gazengel:** data curation, software. **Bruno Marquer:** methodology, investigation. **Juliette Linglin:** methodology, investigation. **Christophe Langrume:** methodology, investigation. **Catherine Porte:** methodology, investigation. **Sylvain Chéreau:** methodology, investigation, writing – review and editing. **Didier Fouville:** methodology, investigation. **Sylvain Fournet:** conceptualization, writing – review and editing, methodology, investigation. **Lionel Lebreton:** conceptualization, methodology, investigation, formal analysis, software, writing – original draft. **Josselin Montarry:** conceptualization, investigation, methodology, funding acquisition, supervision, project administration, writing – original draft.

## Funding

This work was supported by Agence Nationale de la Recherche, ANR‐21‐ECOM‐0003.

## Conflicts of Interest

The authors declare no conflicts of interest.

## Supporting information


**Figure S1:** Effects of rotation crops, root exudate treatments and conditions of exudate application on hatching of *Heterodera carotae* juveniles.
**Figure S2:** Principal Component Analysis (PCA) of soil physico‐chemical properties.
**Figure S3:** Hatching induction of sequential and mixed leek and carrot exudates.
**Figure S4:** Microbial community composition differs between the two types of soil.
**Figure S5:** Description of the soil microbial communities.
**Figure S6:** Microbial community composition is structured by the rotation crop and persists after carrot root exudate application.
**Figure S7:** Microbial community composition at T3 (“soil legacy” effect).
**Figure S8:** Correlation between microbial composition and hatching induction.
**Figure S9:** Comparison of ASV_2 and ASV_144 abundance in the two soils and three rotation crops.

**Table S1:** Physicochemical values for the two soils.
**Table S2:** Mix composition and amplification programs for the two PCR steps of library construction.
**Table S3:** Hatching induction of sequential and mixed leek and carrot exudates.
**Table S4:** DeSeq bacterial analyses.
**Table S5:** DeSeq fungal analyses.
**Table S6:** Correlations between ASV abundances and hatching rate.

## Data Availability

Raw data for this study have been deposited in the European Nucleotide Archive (ENA) at EMBL‐EBI under accession number PRJEB83721 (https://www.ebi.ac.uk/ena/browser/view/PRJEB83721). Accession numbers of soil samples range from ERS22633909 to ERS22634484. All data and R scripts used in this article are available at data.gouv.fr (https://doi.org/10.57745/WL5DJD).
