# Supplementary material for: Variations in Soil Microbiome Assembly Determine Hatching Induction of the Carrot Cyst Nematode Heterodera carotae
Source: Environ Microbiol. 2026 Oct 18;28(10):e70457. doi: 10.1111/1462-2920.70457 (PMC13677158; doi:10.1111/1462-2920.70457)
Supplement: Supplementary file 1 [file EMI-28-e70457-s001.docx]

**Supporting information**

Article: Variations in soil microbiome assembly determine hatching induction of the carrot cyst nematode *Heterodera carotae*

Authors: Marine Biget, Nathan Vannier, Kévin Gazengel, Bruno Marquer, Juliette Linglin, Christophe Langrume, Catherine Porte, Sylvain Chéreau, Didier Fouville, Sylvain Fournet, Lionel Lebreton and Josselin Montarry

The following Supporting Information is available for this article:

**Fig. S1** Effects of rotation crops, root exudate treatments and conditions of exudate application on hatching of *Heterodera carotae* juveniles.

**Fig. S2** Principal Component Analysis (PCA) of soil physico-chemical properties.

**Fig. S3** Hatching induction of sequential and mixed leek and carrot exudates.

**Fig. S4** Microbial community composition differs between the two types of soil.

**Fig. S5.** Description of the soil microbial communities.

**Fig. S6.** Microbial community composition is structured by the rotation crop and persists after carrot root exudate application.

**Fig. S7.** Microbial community composition at T3 (“soil legacy” effect).

**Fig. S8.** Correlation between microbial composition and hatching induction.

**Fig. S9.** Comparison of ASV_2 and ASV_144 abundance in the two soils and three rotation crops.

***Supporting tables are grouped in a same excel document***

**Table S1** Physicochemical values for the two soils.

**Table S2** Mix composition and amplification programs for the two PCR steps of library construction.

**Table S3** Hatching induction of sequential and mixed leek and carrot exudates.

**Table S4** DeSeq bacterial analyses.

**Table S5** DeSeq fungal analyses.

**Table S6** Correlations between ASV abundances and hatching rate.

**Figure S1. Effects of rotation crops, root exudate treatments and conditions of exudate application on hatching of *Heterodera carotae* juveniles.**

Boxplots representing the mean values of the J2 remaining in the cysts as a hatching proxy (mean values ± SEM) at the end of the experiment Tf (after 40 days) for the sandy soil (soil 1) (a) and the muddy soil (soil 2) (b). Three treatments were applied: control (water), D2 (low dose of root exudates) and D1 (high dose of root exudates), either for the bare soil or the soil planted with the rotation crop “planted soil” (condition of exudate application) and the three rotation crops: leek (green), turnip (pink), barley (yellow).

Greeks letters represent significant differences between the conditions of application nested in the factor rotation crop (soil 1: *p* = 4.7e-4; soil 2: n.s.); Capital letters represent significant differences between the treatments within a condition of application (soil 1 : *p* = 2.2 e-16; soil 2 : *p* = 3.9 e-16) and letters among every combination for a soil (linear model with the factor “condition of application” nested in the factor “rotation crop”) (***: *p* < 0.001; ns: *p* > 0.05).

**Figure S2. Principal Component Analysis (PCA) of soil physico-chemical properties.**

The PCA was performed using eight physico-chemical parameters: nitrogen (N), limestone (CaCO3), Carbon (C), organic carbon (TOC), C/N ratio, organic matter (OM), pH and phosphorus (P). Axis 1 explained 99.5% of the variation and corresponds to the differentiation between the two soils (soil1 vs. soil2); the time-point (T0, T3 and Tf), the rotation crop (leek, turnip and barley), the treatment (high dose, low dose and water control) and the condition of application (bare soil or planted soil) have no effect on soil physico-chemical properties.

**Figure S3. Hatching induction of sequential and mixed leek and carrot exudates.**

Dynamic curves (i) representing the cumulated hatched *Heterodera carotae* juveniles across time and boxplot at the end of the experiment (d28) (ii) for (a) sequential and (b) mixed supply of root exudates (leek and carrot). Exudates were supplied 7 times every 4 days. Water condition represents water supply in which no hatching is expected (negative control). (a) The first exudate supply is represented by the first name in the legend. The second one has been supplied 6 times. (b) Proportion (0 to 1) of each exudate (or water control) is provided in the legend. (i) Data points represent mean values of 12 replicates at each time-point. Percentages of hatching is provided on the right for each curve condition (number of hatched juveniles compared to total number of juveniles present in the cysts). (ii) Data at the end of the experiments were analyzed using a Kruskal-Wallis non-parametric tests: (a) sequential *p* = 1.0e-4, n=84; (b) mixed p = 1.0e-4, n=72 (***: *p* < 0.001). Pairwise comparisons (Dunn test with FDR corrections for P-values) are detailed in the Tab. S3.

**Figure S4. Microbial community composition differes between the two types of soil.**

Principal coordinate analysis (PCoA - Bray Curtis index) plots depicted the bacterial (a) and fungal (b) communities (ASV composition) of the two soils at Tf. Results from the permutation tests testing the effect of the soil and the R^2^, percentage of variance explained, are included on the plots (***: *p* < 0.001). The three rotation crops are represented by three colors: leek (green), turnip (pink), barley (yellow).

**Figure S5. Description of the soil microbial communities.**

Composition of the bacterial (a,b,c,d) and fungal (e,f,g,h) communities within the two soils (sandy soil – soil 1 and muddy soil – soil 2) at different time-points (T0, T3 and Tf), conditions of root application (bare soil and soil planted with rotation crops) and treatments (high dose - D1, low dose - D2 and water control). Barplots represent relative abundances at the phylum level (bateria: a, b, c - fungi: e, f, g) or the class level (bacteria: d i, ii, iii - fungi: h i, ii, iii).

**Figure S6. Microbial community composition is structured by the rotation crop and persists after carrot root exudate application.**

Principal coordinate analysis (PCoA - Bray Curtis index) plots depicted the bacterial (a,b) and fungal (c,d) communities (ASV composition) of the two soils at Tf: the sandy soil (soil 1) (a,c) and the muddy soil (soil 2) (b,d). Results from the permutation tests (permanova) testing the rotation crop (r_crop - barley, leek, turnip), the condition of root exudate application (cond. - bare soil, soil planted with the rotation crop “planted soil”) and the treatment (high (D1) and low (D2) doses, water control) effects and the R^2^, percentage of variance explained, are included on the plots (**: *p* < 0.01; ***: *p* < 0.001; ns: *p* > 0.05). The three rotation crops are represented by three colors: leek (green), turnip (pink), barley (yellow).

**Figure S7. Microbial community composition at T3 (“soil legacy” effect).**

Principal coordinate analysis (PCoA - Bray Curtis index) plots depicted the bacterial (a,b) and fungal (c,d) communities (ASV composition) of the two soils between T0 and T3: the sandy soil (soil 1) (a,c) and the muddy soil (soil 2) (b,d). Results from the permutation tests (permanova) testing the time-point effect between T0 and T3 and the rotation crop (r_crop) effect at T3 and the R^2^, percentage of variance explained, are included on the plots (*: *p* < 0.05; **: *p* < 0.01; ***: *p* < 0.001). Square and round dots represent T0 and T3 time-points, respectively. The three rotation crops are represented by three colors: leek (green), turnip (pink), barley (yellow).

**Figure S8. Correlation between microbial composition and hatching induction.**

Linear regressions between bacterial (a) and fungal (b) sample PCoA coordinates (axis 1) and hatching in the sandy soil (soil 1) at the high exudate dose (D1) (condition in which significant variations in hatching have been observed). Linear regressions between bacterial (c) and fungal (d) sample PCoA coordinates (axis 2) and hatching in the muddy soil (soil 2). P-value, and R^2^ were obtained from ANOVA (**: *p* < 0.01; ns: *p* > 0.05).

**Figure S9. Comparison of ASV_2 and ASV_144 abundance in the two soils and three rotation crops.**

Boxplots representing the mean values (± SEM) of the of ASV_2 and ASV_144 relative abundance in the sandy soil (soil 1) (a) and the muddy soil (soil 2) (b) for each rotation crop at the end of the experiment Tf. The P-values and statistics obtained from an ANOVA are indicated in the tables under each graph. The effect of the rotation crop and the soil were tested, as well as the interaction between the two factors.
